# Association between single nucleotide polymorphisms in the PI3K/AKT/mTOR pathway and bladder cancer risk in a sample of Iranian population

**DOI:** 10.17179/excli2017-329

**Published:** 2018-01-02

**Authors:** Fatemeh Bizhani, Mohammad Hashemi, Hiva Danesh, Akbar Nouralizadeh, Behzad Narouie, Gholamreza Bahari, Saeid Ghavami

**Affiliations:** 1 Cellular and Molecular Research Center, Zahedan University of Medical Sciences, Zahedan, Iran; 2 Department of Clinical Biochemistry, School of Medicine, Zahedan University of Medical Sciences, Zahedan, Iran; 3 Urology and Nephrology Research Center; Department of Urology, Shahid Labbafinejad Medical Center, Shahid Beheshti University of Medical Sciences, Tehran, Iran; 4 Department of Human Anatomy and Cell Science, College of Medicine, Faculty of Health Sciences, University of Manitoba, Winnipeg, MB R3E 0J9, Canada; 5 Health Policy Research Center, Shiraz University of Medical Sciences, Shiraz, Iran

**Keywords:** PIK3CA, AKT1, mTOR, polymorphism, bladder cancer

## Abstract

In the past few years several investigations have focused on the role of PI3K/AKT/mTOR pathway and its deregulations in different cancers. This study aimed to examine genetic polymorphisms of this pathway in bladder cancer (BC). In this case-control study, 235 patients with pathologically confirmed bladder cancer and 254 control subjects were examined. *PIK3CA*, *AKT1* and *mTOR* variants were analyzed using polymerase chain reaction-restriction fragment length polymorphism (PCR-RFLP). The findings proposed that the *PIK3CA* rs6443624 SNP significantly decreased the risk of BC (OR=0.44, 95 % CI=0.30-0.65, p<0.0001 CA vs CC; OR=0.35, 95 % CI=0.16-0.78, p=0.0107, AA vs CC; OR=0.60, 95 % CI=0.46-0.79, p=0.0002, A vs T). The *AKT1* rs2498801 variant is associated with a decreased risk of BC (OR=0.57, 95 % CI=0.39-0.82, p=0.003, AG vs AA; OR=0.74, 95 % CI=0.56-0.97, p=0.032, G vs A) while, AKT1 rs1130233 polymorphism considerably increased the risk of BC (OR=3.70, 95 % CI=2.52-5.43, p<0.0001, GA vs GG; OR=5.81, 95 % CI=1.53-21.97, p=0.010, AA vs GG; OR=2.71, 95 % CI=1.98-3.70, p<0.0001, A vs G). Additionally, mTOR rs2295080 variant notably increased the risk of BC (OR=2.25, 95 % CI=1.50-3.38, p<0.0001, GT vs GG; OR=4.75, 95 % CI=2.80-8.06, p<0.0001, TT vs GG; OR=3.10, 95 % CI=2.34-4.10, p<0.0001, T vs G). None of the other examined polymorphisms (*AKT1* rs1130214, *AKT1* rs3730358, *mTOR* rs1883965) revealed significant association with BC. In conclusion, our findings suggest that *PIK3CA* rs6443624, *AKT1* rs2498801, *AKT1* rs1130233, as well *mTOR* rs2295080 polymorphism may be related to bladder cancer development in a sample of Iranian population. Validation of our findings in larger sample sizes of different ethnicities would provide evidence on the role of variants of PI3K/AKT/mTOR pathway in developing BC.

## Introduction

Cancer is a major global public health problem (Siegel et al., 2016[41]). Bladder cancer is among the most common types of cancer in the world, and a recent study reported 429,800 new cases diagnosed with bladder cancer in the year 2012 and a fatality rate of 165,100 (Torre et al., 2015[44]). It has been suggested that in addition to environmental risk factors such as obesity, smoking, and physical inactivity (Burger et al., 2013[5]; Shiels et al., 2014[40]; Vermeulen et al., 2015[45]), genetic factors contribute to bladder cancer development (Aben et al., 2002[1]; Giedl et al., 2016[16]; Hua et al., 2016[22]; Sankhwar et al., 2016[38]). 

One of the main features of cancer development and progression is shifting of metabolism balance toward faster and higher energy production to support energy demand for highly proliferative cancer cells (Massari et al., 2016[31]). It has been previously reported that many metabolic pathways are changed and might be involved in BC tumorigenesis and malignancies. Recent efforts have been focused on identifying these pathways and their potential applications as serum specific biomarkers in early diagnosis (Griffin and Shockcor, 2004[17]). The phosphatidylinositol 3-kinase (PI3K)/AKT/mTOR is involved in regulation of several basic cellular mechanisms comprising cell growth, cell survival, cell motility, angiogenesis, as well as cell metabolism (Knowles et al., 2009[25]; Courtney et al., 2010[12]; Bartholomeusz and Gonzalez-Angulo, 2012[3]; McCubrey et al., 2012[32]). Recent investigations have revealed that mutations in *PIK3CA* deliberate sensitivity to AKT targeted therapy in bladder cancer by regulating DUSP1 expression and subsequent ERK1/2 dephosphorylation and can potentially serve as a stratifying biomarker for treatment (Sathe et al., 2014[39]).

PI3K has two different subunits which is involved in regulation of its activity (p85) and its catalytic function (p110) (Guerrero-Zotano et al., 2016[18]).* PIK3CA* gene is located on chromosome 3q26.3 that encodes the p110alpha catalytic subunit of phosphoinositide 3-kinases (PI3Ks) (Karakas et al., 2006[23]). PI3Ks serve as oncogenes and belong to a conserved family of lipid kinases that phosphorylate the 3'-hydroxyl group of phosphoinositides and produce phosphatidylinositol-3,4,5-trisphosphate (PIP3), a critical second messenger that recruits AKT for activation of growth, proliferation and survival signaling (Cantley, 2002[6]). It has been proposed that mutations in the *PIK3CA* gene is occurred approximately in 15 % of human cancers (Karakas et al., 2006[23]). mTOR inhibitors for prostate cancer patients with TSC1 (tuberous sclerosis complex 1) mutations and concomitant targeting of PI3K and MEK represent approaches to block PI3K/AKT/mTOR pathway (Carneiro et al., 2015[7]).

The *AKT1* gene has been mapped to human chromosome 14 (14q32) (Staal et al., 1988[42]). *AKT* proto-oncogen is a serine/threonine kinase with three isoforms (AKT1, AKT2, and AKT3). It is a downstream target of the PI3K and plays an important role in cancer cell survival, cell cycle entry, and glucose metabolism (Engelman, 2009[14]). Polymorphisms of *AKT* gene has been shown to be associated with various cancers including nasopharyngeal carcinoma (NPC) (Zhang et al., 2014[51]), gastric cancer (GC) (Piao et al., 2015[34]), and prostate cancer (Chen et al., 2012[8]). Korkolopoulou et al. (2012[26]) proposed that PIK3CA/AKT1 mutational status could be a potential predictive marker for time-to-recurrence of bladder urothelial carcinoma.

The mammalian target of rapamycin (mTOR) gene is mapped to chromosome 1 (1p36.22). mTOR is a serine/threonine kinase that functions as a downstream effector of the PI3K/AKT signaling pathway. It exists as two multiprotein complexes, mTORC1 and mTORC2 (Keppler-Noreuil et al., 2016[24]). Hyper-activation of the PI3K/AKT/mTOR pathway results in substantial dysregulation of normal cellular functions, which may lead to various human cancer development (Yuan and Cantley, 2008[49]; Knowles et al., 2009[25]; Platt et al., 2009[35]; Porta et al., 2014[36]; Houede and Pourquier, 2015[21]; Tan et al., 2015[43]; Yuge et al., 2015[50]; Guerrero-Zotano et al., 2016[18]). 

There is little information regarding the effects of PI3K/AKT/mTOR pathway polymorphisms on bladder cancer (Chen et al., 2009[10]; Lin et al., 2010[28]). To the best of our knowledge there is no data concerning the impact of PI3K/AKT/mTOR polymorphism on the risk of developing cancer in the Iranian population. Therefore, this case-control study was designed to assess the possible association between *PIK3CA*, *AKT1* and *mTOR* polymorphisms and susceptibility to bladder cancer in an Iranian population. 

## Materials and Methods

### Patients

The current case-control study has been done in a population of 235 confirmed bladder cancer patients and 254 sex and age matched cancer free subjects as the control group. Demographic and clinicopathological characteristics of bladder cancer patients and controls are shown in Table 1(Tab. 1). The study protocol for recruitment was approved by the local Ethics Committee of Zahedan University of Medical Sciences (IR.ZAUMS.REc.1394. 325), and informed consent was obtained from all patients and healthy individuals. 

### Genotyping

Genotyping of the variants was done using polymerase chain reaction-restriction fragment length polymorphism (PCR-RFLP) methods. The primers sequences are listed in Table 2(Tab. 2). 

**Figure 1:**
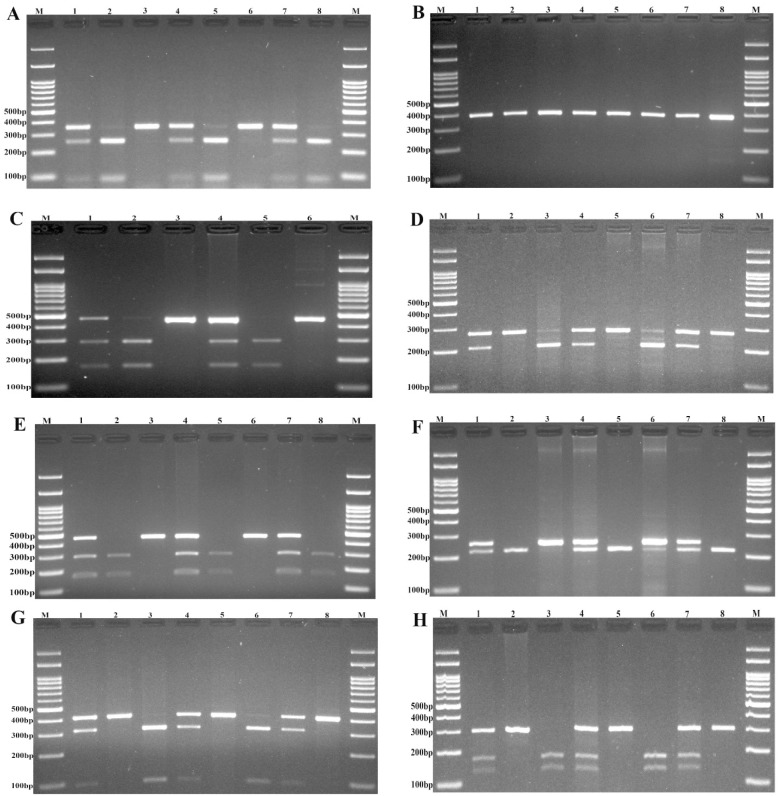
Photograph of electrophoresis pattern of the PCR-RFLP method for detection of PIK3CA rs6443624 (A), PIK3CA rs141178472 (B), AKT1 rs2498801 (C), AKT1 rs1130233 (D), AKT1 rs1130214 (E), AKT1 rs3730358 (F), mTOR rs1883965 (G), and mTOR rs2295080 (H). M: DNA marker. For PIK3CA rs6443624, lanes 1, 4, and 7: CA; lanes 2, 5, and 8: CC; lanes 3, and 6: AA. For PIK3CA rs141178472, all lanes are TT. For AKT1 rs2498801, lanes 1, and 4: AG; lanes 2, and 5: GG; lanes 3, and 6: AA. Regarding AKT1 rs1130233, lanes 1, 4, and 6: GA; lanes 1, 5, and 8: GG; lanes 3, and 6: AA. For AKT1 rs1130214, lanes 1, 4 and 7: TG; lanes 2, 5, and 8: GG; lanes 3, and 6: TT. For AKT1 rs3730358, lanes 1, 4, 6, and 7: TC; lanes 2, 5, and 8: CC; lane 3: TT. For mTOR rs1883965, lanes 1, 4, and 7: AG; lanes 2, 5, and 8: GG; lanes 3, and 6: AA. For mTOR rs2295080, lanes 1, 4, and 7: TG; lanes 2, 5, and 8: GG; lanes 3, and 6: TT.

1 μl genomic DNA (~100 ng/μl), 1 μl (10 μM) forward and reverse primers, 10 μl 2X Prime Taq Premix (Genet Bio, Korea), and 7 μl ddH2O were added into a 0.20 ml PCR reaction tube. The PCR conditions were, initial denaturing step at 95 °C for 5 min followed by 30 cycles of denaturation at 95°C for 30 s, annealing at appropriate temperature (Table 1(Tab. 1)) for 30 s, extension at 72 °C for 30 s, and then a final extension step for 10 min at 72 °C. Then, 10 μl of PCR product was digested by suitable restriction enzyme (Table 1(Tab. 1)) based on the manufacturer's procedure (Figure 1(Fig. 1)). The digested products were electrophoresed on agarose gel containing 0.5 μg/ mL ethidium bromide, visualized on a UV transilluminator and photograph was taken (Figure 1(Fig. 1)). For the quality control of genotyping; approximately, 20 % of the random samples were blindly regenotyped and the reproducibility was 100 %.

### Statistical analysis

All statistical analyses were done using statistical package SPSS 22 software. The categorical and continuous variables were examined using χ2 and t-test, respectively. The association between genotypes and BC were evaluated by computing the odds ratio (OR) and 95 % confidence intervals (95 % CI) from non-conditional logistic regression analyses. The p-values less than 0.05 were considered as statistically significant. 

## Results

The study group consisted of 235 bladder cancer patients (193 males, 40 females; age 63.4 ± 12.1 years) and 255 cancer free subjects as control (210 males, 42 females; age 62.3 ± 10.7 years). No significant difference was observed between the groups regarding sex and age (p=0.904 and p=0.316, respectively). 

The genotype and allele frequencies of polymorphism in bladder cancer patients and control group are shown in Table 3(Tab. 3). The findings proposed that the PIK3CA rs6443624 SNP significantly decreased the risk of BC (OR=0.44, 95 % CI=0.30-0.65, p<0.0001 CA vs CC; OR=0.35, 95 % CI=0.16-0.78, p=0.0107, AA vs CC; OR=0.60, 95 % CI=0.46-0.79, p=0.0002, A vs T). The AKT1 rs2498801 variant is associated with a lower risk of BC (OR=0.57, 95 % CI=0.39-0.82, p=0.003, AG vs AA; OR=0.74, 95 % CI=0.56-0.97, p=0.032, G vs A). While, AKT1 rs1130233 polymorphism significantly increased the risk of BC (OR=3.70, 95 % CI=2.52-5.43, p<0.0001, GA vs GG; OR=5.81, 95 % CI=1.53-21.97, p=0.010, AA vs GG; OR=2.71, 95 % CI=1.98-3.70, p<0.0001, A vs G). In addition, mTOR rs2295080 variant significantly increased the risk of BC (OR=2.25, 95 % CI=1.50-3.38, p<0.0001, GT vs GG; OR=4.75, 95 % CI=2.80-8.06, p<0.0001, TT vs GG; OR=3.10, 95 % CI=2.34-4.10, p<0.0001, T vs G). None of the other examined polymorphisms (AKT1 rs1130214, AKT1rs3730358, mTOR rs1883965) revealed significant association with BC.

## Discussion

*PI3K*, *AKT* as well as *mTOR* are proto-oncogenes. Genetic variants in PI3K/AKT/ mTOR pathway may affect critical cellular functions and increase an individual's cancer risk (Mahdi et al., 2015[30]). Mutations of PI3K/AKT/mTOR pathways are frequently found in cancer, particularly breast cancer where about 60 % of tumors harbor genetic alterations that hyperactive this signaling pathway and was found to be associated with cellular transformation, carcinogenesis and drug resistance (Engelman, 2009[14]; Guerrero-Zotano et al., 2016[18]). Mounting evidences proposed that the PI3K/AKT/mTOR pathways are generally activated in many cancers including bladder cancer, and inhibitors of these core genes are displaying great promise as the latent anticancer agents (Bellacosa et al., 2005[4]; Bartholomeusz and Gonzalez-Angulo, 2012[3]). 

In the present study we investigated the impact of *PI3K/AKT/mTOR* gene polymorphisms on bladder cancer risk. The findings revealed that *PIK3CA* rs6443624 C>A and *AKT1* rs2498801A>G variants caused a significant decline in the risk developing bladder cancer. The rs1130233 G>A variant of *AKT1* and rs2295080 G>T variant of mTOR significantly increased the risk of BC. While, we detected no significant association between AKT1 rs1130214 T>G and rs3730358 C>T variants, as well as mTOR rs1883965 G>A variant and risk of bladder cancer. 

It has been shown that *PIK3CA* rs7646409 variant increased the risk of osteosarcoma in the Chinese population (He et al., 2013[20]). Ding et al. (2015[13]) proposed that a miR-520a binding site polymorphism rs141178472 in the *PIK3CA* 3′-untranslated region (3′-UTR) increased the risk of development colorectal cancer. Chen et al. (2009[10]) have investigated germ line genetic variations in the PI3K-AKT-mTOR pathway and bladder cancer risk. They found that four SNPs (rs11653499 G>A, rs7211818 A>G, rs7212142 A>G, and rs9674559 A>G) in *RAPTOR* significantly associated with increased bladder cancer risk. While no significant correlation between rs12045585 and rs2994329 variants of AKT3 and bladder cancer risk was observed (Chen et al., 2009[10]). It has been shown that three SNPs in PI3K-AKT-mTOR pathway (AKT2 rs3730050, PIK3R1 rs10515074, and RAPTOR rs9906827) were significantly associated with survival in invasive and metastatic bladder cancer patients (Chen et al., 2010[11]). Lin et al. (2010[28]) revealed that high caloric intake and low physical activity conferred increased bladder cancer risk and that the risk may be influenced by genetic variants of PI3K/AKT/mTOR pathway genes. 

The expression of *PIK3CA* was found to be increased in human papillary thyroid carcinoma (PTC) tissue and microRNA-363-3p, as a tumor suppressor gene inhibits PTC progression by targeting PIK3CA (Liu et al., 2017[29]). It has been shown that miR-490-5p suppresses tumor growth in renal cell carcinoma by binding to the 3'-UTR of the *PIK3CA* mRNA and reduce the expression of PIK3CA at both mRNA and protein levels, which further inhibits phosphatidylinositol 3-kinase/Akt signaling pathway (Chen et al., 2016[9]).

Fallah et al. (2015[15]) did not find any significant association between AKT1 rs72715985, rs2494750 and rs74090038 variants and risk of endometrial cancer in a sample of Iranian population. Zhu et al. (2016[52]) reported that none of the AKT1 (rs2494750, rs2494752 and rs10138277) and AKT2 (rs7254617 and rs2304186) variants showed an association with esophageal squamous cell carcinoma (ESCC) risk in an Eastern Chinese population. Wang et al. (2016[47]) examined the impact of *AKT1* rs2494750 G>C, rs2494752A >G, rs10138227C >T polymorphisms as well as *AKT2* rs7254617 G>A and rs2304186G >T variants on gastric cancer. They found that only *AKT1* rs2494752 variant significantly increased the risk of gastric cancer susceptibility, probable by modulating the *AKT1* promoter transcriptional activity (Wang et al., 2016[47]). It has been shown that rs1130214 and rs3803300 variants of AKT1 significantly increased the risk of OSCC in Chinese Han Population (Wang et al., 2015[48]). 

Chen et al. (2012[8]) have found a significant association between *mTOR* rs2295080 as well as *AKT2* rs7254617 variant and prostate cancer (PCa) risk in a Chinese population. It has been revealed that AKT/mTOR over-expressed and PTEN expression was significantly decreased in conditions of proliferative dysregulation and a variety of solid tumors including prostate cancer and gastric cancer (Nicholson and Anderson, 2002[33]; Hartmann et al., 2009[19]; Riquelme et al., 2016[37]). 

The rs2677764 variant of *PIK3CA* showed a significant association with endometrial cancer (Lacey et al., 2011[27]), while other variants of *PIK3CA* (rs2699905, rs7641889, rs7641983, rs7651265, rs6443624, rs7640662, rs2677760, rs3729692, rs1607237, rs6786049) were not associated with the disease. It has been proposed that PI3K signaling pathway is activated by PIK3CA mRNA over-expression, in prostate cancer (Agell et al., 2011[2]). 

Somatic aberrations of PI3K-AKT-mTOR pathway genes have been generally observed in a variety of malignancies. Targeting the genetic variations of the PI3K/AKT/mTOR pathway has potential use in the treatment of various cancer (Vivanco and Sawyers, 2002[46]; Carneiro et al., 2015[7]; Houede and Pourquier, 2015[21]; Guerrero-Zotano et al., 2016[18]). 

In summary, the findings of the present study provide an association between PI3K-AKT-mTOR pathway gene polymorphisms and risk of developing bladder cancer in a sample of Iranian population. Validation of the findings in larger sample sizes and different ethnicities would provide evidence for the role of variants of PI3K/AKT/mTOR pathway in bladder cancer development.

## Acknowledgement

This paper was funded by a dissertation grant (MSc thesis of FB #7540) from the Deputy for Research, Zahedan University of Medical Sciences. 

## Disclosure of conflicting interests

The authors declare that there is no conflict of interest to disclose.

## Figures and Tables

**Table 1:**
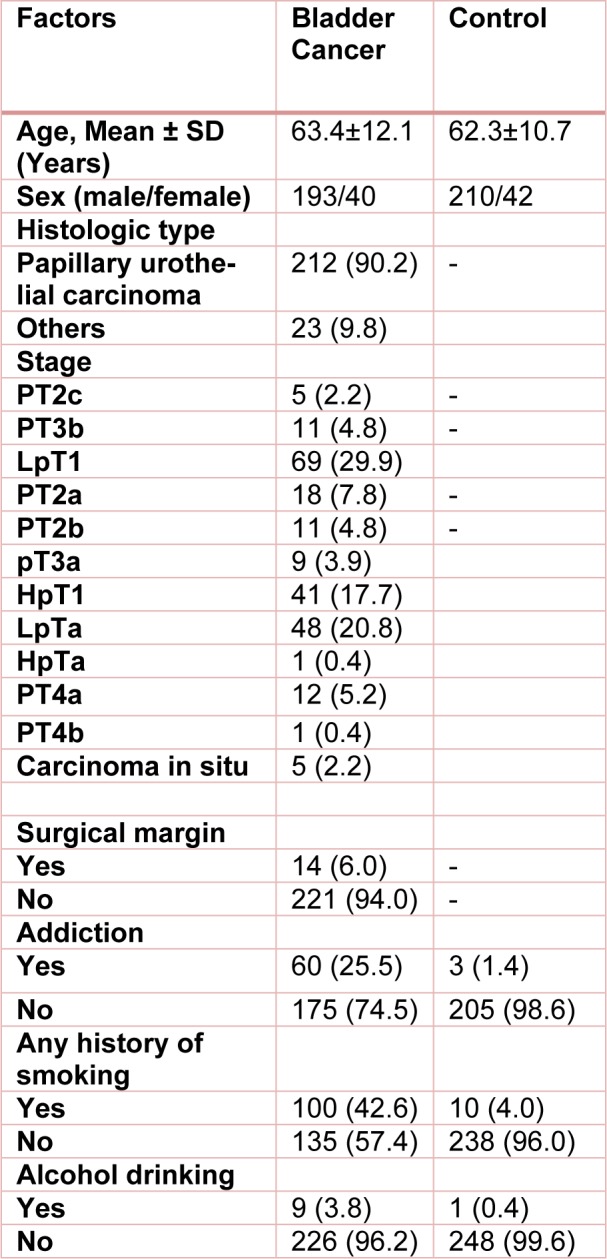
Demographic and clinicopathological characteristics of bladder cancer patients and control subjects

**Table 2:**
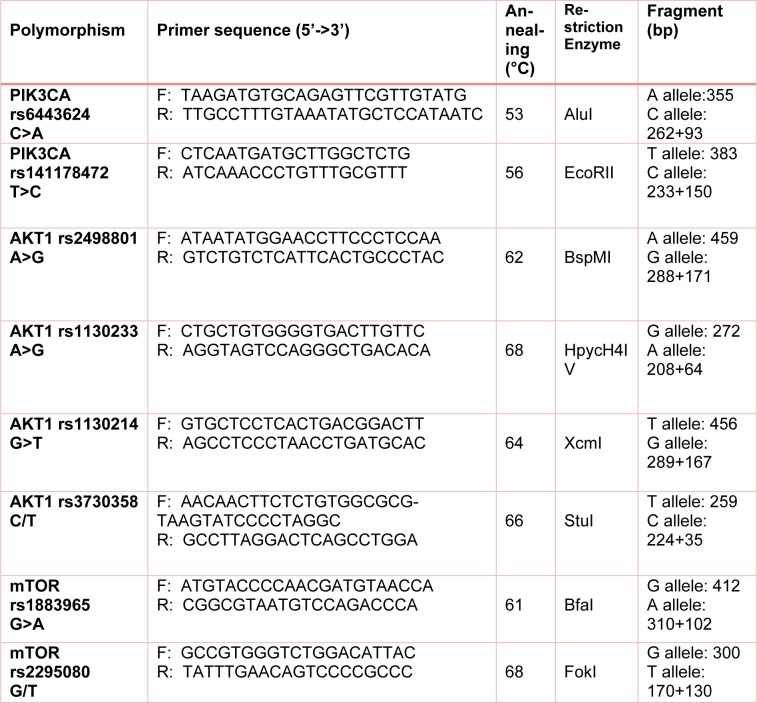
The primers used for detection of PI3K/AKT/mTOR polymorphisms

**Table 3:**
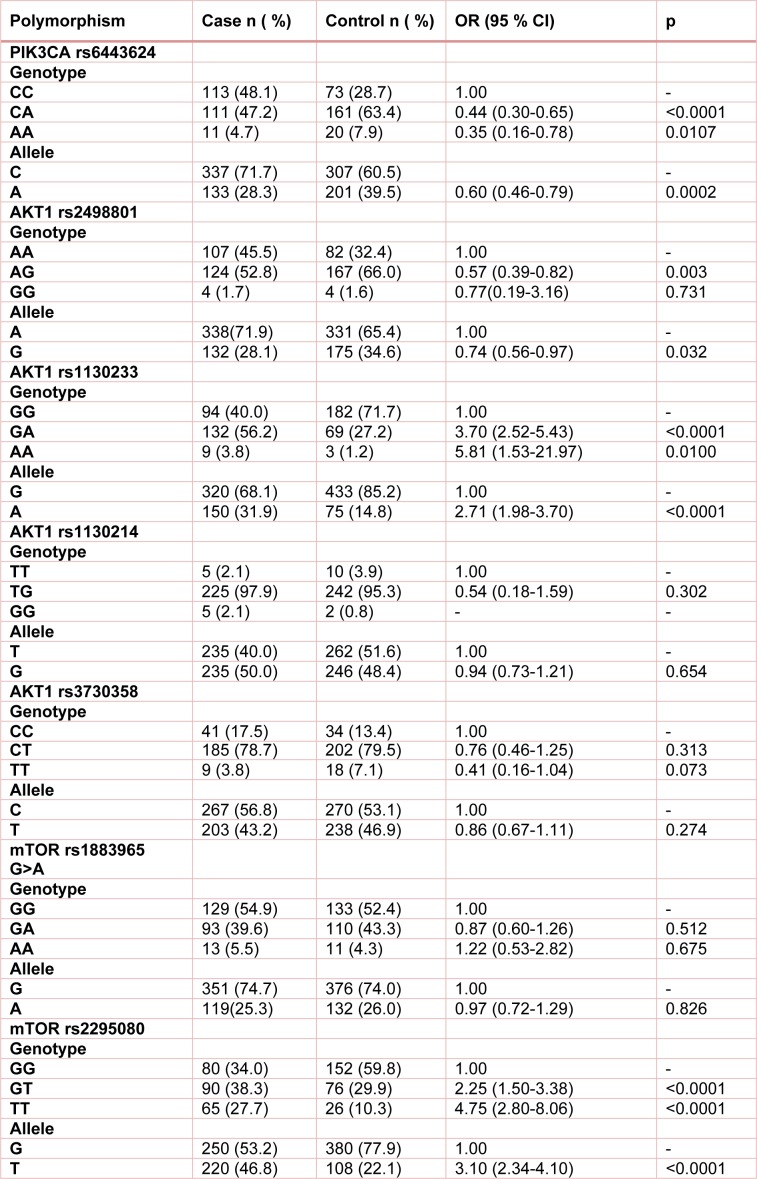
Genotype and allele frequencies of *miRNAs* polymorphisms in bladder cancer and controls
